# Synergy Factorized Bilinear Network with a Dual Suppression Strategy for Brain Tumor Classification in MRI

**DOI:** 10.3390/mi13010015

**Published:** 2021-12-23

**Authors:** Guanghua Xiao, Huibin Wang, Jie Shen, Zhe Chen, Zhen Zhang, Xiaomin Ge

**Affiliations:** 1 College of Computer and Information Engineering, Hohai University, Nanjing 211100, China; smoothxiao@hhu.edu.cn (G.X.); shenjie_2003045@hhu.edu.cn (J.S.); chenzhe@hhu.edu.cn (Z.C.); zz_hhuc@hhu.edu.cn (Z.Z.); 2 Department of Equipment Engineering, Jiangsu Urban and Rural Construction College, Changzhou 213147, China; 3 Department of Radiology, Changzhou Second People’s Hospital Affiliated to Nanjing Medical University, Changzhou 213000, China; gexiaomin082@126.com

**Keywords:** brain tumor classification, convolutional neural networks, suppressing strategy, bilinear pooling, magnetic resonance imaging

## Abstract

Automatic brain tumor classification is a practicable means of accelerating clinical diagnosis. Recently, deep convolutional neural network (CNN) training with MRI datasets has succeeded in computer-aided diagnostic (CAD) systems. To further improve the classification performance of CNNs, there is still a difficult path forward with regards to subtle discriminative details among brain tumors. We note that the existing methods heavily rely on data-driven convolutional models while overlooking what makes a class different from the others. Our study proposes to guide the network to find exact differences among similar tumor classes. We first present a “dual suppression encoding” block tailored to brain tumor MRIs, which diverges two paths from our network to refine global orderless information and local spatial representations. The aim is to use more valuable clues for correct classes by reducing the impact of negative global features and extending the attention of salient local parts. Then we introduce a “factorized bilinear encoding” layer for feature fusion. The aim is to generate compact and discriminative representations. Finally, the synergy between these two components forms a pipeline that learns in an end-to-end way. Extensive experiments exhibited superior classification performance in qualitative and quantitative evaluation on three datasets.

## 1. Introduction

A brain tumor is an abnormal growth of tissue in the brain or central vertebral canal, which can cause a variety of serious complications such as sensory and motor dysfunction. Brain tumors endanger the lives of patients and it is usually a complex process to diagnose them. The accurate diagnosis of brain tumors in the early stage allows greater opportunity for curing them, since the treatments vary from one type to another. Magnetic resonance imaging (MRI) is considered a non-invasive soft tissue imaging used to help identify brain tumor types. In clinical practice, the inspection of MRI images depends on experienced specialists and radiologists. Manual diagnosis of brain tumors in the face of a torrent of patient data is difficult and the process is hectic and error-prone. Many researchers are committed to computer-aided diagnostic (CAD) systems based on machine learning to automate the diagnostic process, thereby boosting the efficiency of precise analysis decisions and aiding physicians by enhancing diagnostic capabilities. According to the World Health Organization (WHO) guidelines [1], the classification of brain tumors is strictly histopathology. This classification also promotes the use of CAD in the existing medical imaging technology.

In recent years, researchers have proposed various prediction models for brain tumors. The traditional methods extract statistical features from MRI slices based on the image’s characteristics (texture, boundary, and shape). Feature representations include discrete wavelet transform (DWT) [2], Gabor filter [3], scale invariant feature transform (SIFT) [4], Harris corner [5], etc. Feature reduction methods, such as principal component analysis (PCA) [6], locally linear embedding (LLE) [7], and T-distributed stochastic neighbor embedding (TSNE) [8] are then used to decrease the fused features. Finally, the feature datasets extracted by different methods are passed to an ensemble classifier for classification. Typical classifiers includes K-nearest neighbor (KNN) [9], support vector machine (SVM) [10], latent dirichlet allocation (LDA) [11], random forest (RF) [12], artificial neural network (ANN) [13], etc. These traditional methods benefit from model interpretability and small data size. However, for a long time there was little scope in performance improvements, until deep convolutional neural networks (CNNs) came to dominate. Most traditional methods struggle with prior knowledge about domain expertise, whereas deep CNNs have greatly improved on pattern recognition [14,15,16], due to their excellent performance close to that of humans.

Many previous works have attracted considerable attention in terms of classification, mainly described in the following aspects. Firstly, they have built new deep CNN models according to the properties of datasets and the carrying capacity of computing resources. Ayadi et al. [17] aimed to build a CNN model containing various layers to classify MRI brain tumors with little preprocessing. Khan et al. [18] introduced a scratched CNN model and combined it with data augmentation and image processing to categorize MRI brain scan images. Bashir-Gonbadi et al. [19] used a convolutional auto-encoder neural network (CANN) to extract desirable features for brain tumor classification. Alhassan et al. [20] adopted an activation function with a hard swish-based RELU in CNNs to improve classification performance and learning speed. Secondly, previous studies have utilized pre-trained models to transfer knowledge and model weights in medical image datasets. Polat et al. [21] conducted classic CNN architectures with different optimization algorithms to explore the transfer learning technique. Bodapati et al. [22] integrated two deep CNN architectures to allow joint training sets of image representations in an end-to-end manner. Sajjad et al. [23] put augmented datasets into a segmentation network and used a pre-trained CNN model to obtain convincing performance. Swati et al. [24] introduced a general deep CNN model with a block-wise fine-tuning strategy based on transfer learning and that does not require any handcrafted features. Thirdly, researchers have stacked ensemble learning to play down error factors by complementing each model. Toğaçar et al. [25] developed a CNN model called BrainMRNet for mass detection of MRI images that includes attention modules, hypercolumn technique, and residual blocks. Anaraki et al. [26] utilized a genetic algorithm (GA) to evolve the CNN model and bagged them as an ensemble algorithm for classification. Kumar et al. [27] offered a weighted correlation feature selection-based iterative Bayesian multivariate deep neural learning (WCFS-IBMDNL) technique to improve early-stage brain tumor diagnosis. Noreen et al. [28] presented a fine-tuned deep CNN model and encompassed the ensemble technique including RF, KNN, and SVM.

The modified CNN models mentioned above have shown favorable results in diverse applications of medical image classification. However, there are still challenges for networks to precisely understand differences in order to handle inter-class similarities and intra-class variations. Firstly, deep CNNs provide the capability to make predictions and draw conclusions by learning data feature representations. This pattern tends to fail when faced with the challenge of limited training data for the over-fitting problem, which means that deep learning is a data starving approach. Transfer learning is a well-established technique that has performed successfully in dramatically improving the results of CNN models, especially under the conditions associated with a relatively small dataset and low computational power. Transfer learning also has flaw of paying little attention to intermediate feature representations generated by the pre-trained CNN model. Secondly, deep CNN-based models classify by learning data feature mappings between input images and output labels. Common convolutional layers of CNNs act as sliding windows, resulting in output feature maps generated with relative spatial arrangement. This tendency during learning causes CNN models to focus on local spatial information of brain tumors and to lack attention to the relationship between tumors and other tissues in an MRI brain image. Recent CNN models such as FV-CNN [29], NetVLAD [30], and Deep-TEN [31] perform an outstanding job of extracting global orderless features for material or texture recognition. However, this statistical- and distribution-based acquisition approach may distract attention from salient features in brain tumor identification, since almost all MRI brain images have a very similar background and texture. Thirdly, the CNN architecture implements the final prediction by aggregating output features from various convolutional channels. Usual aggregation practices, such as concatenation, pooling, and element-wise sum, can only collect first-order statistical information, which cannot contribute to the classification of MRI brain tumor images. This is because brain tumor classification is essentially a fine-grained recognition task and requires more detailed visual descriptions. In addition, despite ensemble learning being a beneficial strategy in improving the efficiency and stability of CNNs, these models require more computation to combine the predictions in test time. Furthermore, the model pipeline is not always learned in an end-to-end scenario and is probably not the best option for real-time applications.

To address all the previous concerns, we propose to guide the network to find exact differences among similar classes. We first present a customized dual suppression encoding (DSE) block that gears our network to refine global orderless information and local spatial representations. According to the suppression strategy for global orderless features, the aim is to reduce the negative salient features’ impact based on background and texture, and guide the network to concentrate on informative ones for better discriminability. The suppression strategy for local spatial features does the opposite during learning. It extends the network attention to salient parts to consolidate a diverse set of relevant cues across the classes. Then, we introduce a fusion layer based on improved bilinear pooling [32] to fuse the extracted features, which refer to a factorized bilinear encoding (FBE) layer. Unlike linear pooling and bilinear pooling, FBE avoids high-dimensional outputs and produces huge model parameters. It also enjoys the benefit of robust expressive capacity, allowing it to obtain a compact and discriminative representation with fewer parameters. Finally, we designed a framework to plug these two blocks (DSE and FBE) into an existing state-of-the-art deep CNN to form an end-to-end deep learning pipeline for our classification task.

The main contributions of our work are four-fold.

- We propose a DSE block that can guide the network to refine the global orderless information and the local spatial representations on purpose.
- We introduce a fusion layer named FBE to fuse the extracted features in a compact and discriminative form, enabling the network to offer more expressive and robust representation.
- We design a framework with transfer learning for end-to-end classification tasks where the DSE block and the FBE layer are learned together in a single network.
- Comparative experiments demonstrate that the proposed model is competent to support the decision-making processes of specialists in clinics.

The organization of the layout in this paper is as follows. Section 2 reviews the related works. Section 3 gives a detailed description of the proposed method. Section 4 provides comprehensive experiments and analysis. Finally, Section 5 draws conclusions and suggestions for future work.

## 2. Related Works

### 2.1. Feature Encoding

The progress of deep CNNs in vision-based applications does not imply that traditional computer vision techniques are obsolete or superseded. Deep CNNs are sometimes unnecessary when dealing with images with imperceptible inter-class variance and wide intra-class variance if the training dataset is not well constructed. In traditional computer vision tasks, image feature extracting is a set of feature descriptors (SIFT, SURF, BRIEF, etc.) followed by feature encoding approaches such as bag of features (BoF) [33], Fisher vector (FV) [34] and vector of locally aggregated descriptors (VLAD) [35]. These features form a bag of visual words (BoVW) to perform the tasks such as image classification. The difficulty with the hand-engineered approach is how to choose the best describable features to distinguish different objects. The features in BoVW become more cumbersome and require more parameters to define as the number of classes increases. Thus, the base intuition behind encoding approaches is to pay more attention to a solution of global features in a specific domain. Some studies embed feature encoding approaches into deep CNNs to achieve improved performance in their domain tasks. Ng et al. [36] adopted VLAD encoding to encode convolutional features extracted from different layers of deep networks for image retrieval tasks. Tang et al. [37] combined CNN training with FV encoding in a single end-to-end structure (FisherNet). They then observed both accuracy and computational efficiency over plain CNN and standard FV on visual object class classification tasks. Inspired by these, we organize our network structure using an encoding block covering VLAD and FV to leverage orderless features.

### 2.2. Feature Fusion

The implementation of feature aggregation from different convolutional channels is often via summation or concatenation. Recent studies [38,39] show that bilinear pooling exhibits more expressive ability among feature fusion approaches by exploiting higher-order information. However, bilinear pooling generates redundant information in an exceptionally high-dimensional space [40], therefore suffering from the burstiness phenomenon [41]. Gao et al. [42] proposed a kernelized bilinear pooling method to reduce the feature dimensionality. The obtained compact representations are comparable to fully bilinear pooling in performance. Li et al. [43] presented a factorized bilinear model (FB) that replaces the pairwise feature with factorized bilinear weight. This model thereby simplifies the calculation to linear complexity. Gao et al. [44] adopted sparse coding and dictionary learning in a fusion technique based on bilinear pooling, named factorized bilinear coding (FBC), to immensely reduce the number of parameters of the model. Although these models provide considerable improvements in related target issues, a compact and discriminative solution of bilinear pooling is still required for our model to be optimized.

### 2.3. Feature Mapping

Classification models based on deep CNN usually consist of two parts: a convolutional layer and a fully connected layer. Multiple convolutional layers comprise the core building block of a deep CNN. Fully connected layers take the features extracted from convolutional layer mapping into the label space. Each layer combines a set of learnable parameters to interpret the input feature. We can formulate this as $y=f(\sum_{i}\omega_{i}x_{i}+b_{i})$, where $x_{i}$ is input data, $\omega_{i}$ and $b_{i}$ denote the weights and the bias term and *f* denotes a non-linear activation function.

**Figure 1:**
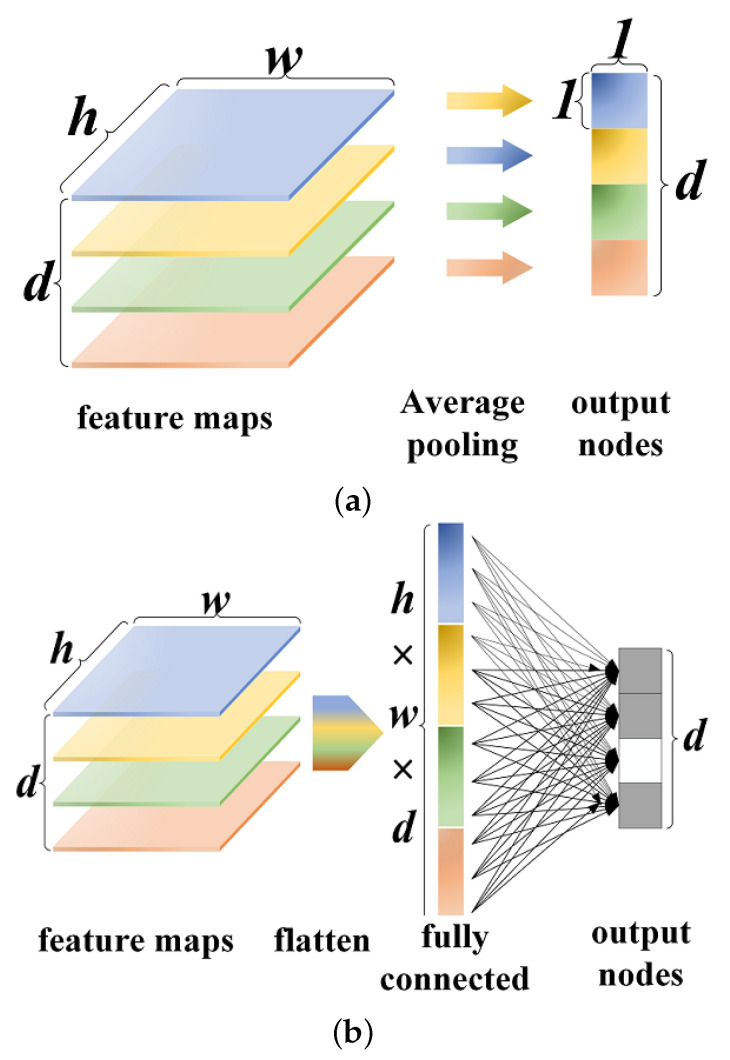
Comparison of feature mapping layers. (**a**) FC layer, (**b**) GAP layer.

Studies using MRI images in [45,46] show that transfer learning may utilize the parameter values of the weights and bias from a pre-trained network to gain generalized features and then fine-tune them on specific datasets of MRI images. This feature-based learning approach does not require training the whole model from scratch in most cases. Inspired by [47], some recent papers on medical images, such as [48], have verified the effectiveness of replacing FC layers in their deep CNNs with GAP to avoid overfitting on the corresponding layers and maintain high performance. Figure 1 sketches the differences between the FC layer and the GAP layer.

The FC layer stretches the feature maps of the convolution layer into a vector, then produces a result with matrix multiplication followed by a bias addition. It requires a mass of trainable parameters to perform complex nonlinear computations in the feature space, making it heavily dependent on dropout regularization and possibly resulting in overfitting. The GAP layer averages each feature map of the convolution layer to create confidence maps of the corresponding category. Endowing each feature map with an actual category meaning makes it more interpretable to the convolution structure. Hence, our work draws on the conception of GAP to reduce the network parameters and then uses FC to generate the desired number of categories.

## 3. Proposed Method

In this section, we present our classification solution in three ways: First, we introduce the overall network architecture of the proposed model. Then, we elaborate on the DSE block. Finally, we explain how the FBE layer works.

### 3.1. Proposed Architecture

**Figure 2:**
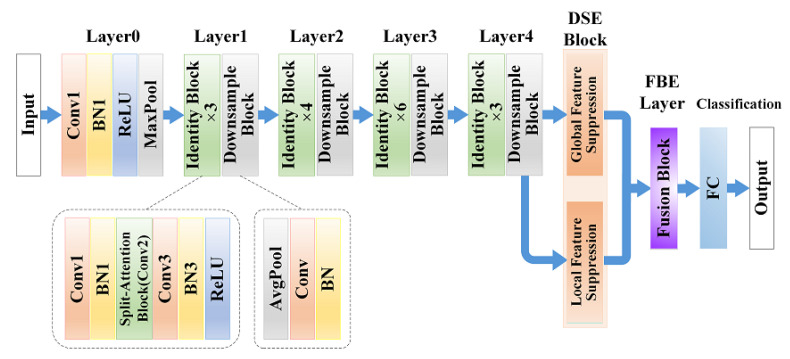
Architecture of the proposed network.

As is illustrated in Figure 2, we introduce the architecture of the brain tumor classification model with the following three parts: backbone network, DSE block, and FBE layer. The primary purpose of this devised framework is to distinguish the subtle features extracted from different MRI images among brain tumor types. As mentioned previously, the transfer learning technique is superior to training a deep CNN from scratch due to limited training data. Feature encoding gives the extracted features a different interpretation, enhancing the model’s expressive ability and generalization for fine-grained features. Drawing on feature encoding, we derive features from convolutional layers with a pre-trained CNN model as a feature extraction module, which provides better details of local feature representations. Then, we feed the outputs from convolutional layers into the DSE block through two paths. One is the original CNN path that encodes the features into spatial information. The other is the residual encoding [31] that sends the copies into encoders such as VLAD and Fisher vector to generate orderless representations. The discriminative regions of the feature maps from these two paths are suppressed separately by customized strategies, with the expectation that our network will look for more representative and informative features to enhance classification performance. Finally, we build the FBE layer based on modified bilinear pooling [32] to capture higher-order information. This immensely reduces the number of parameters to avoid overfitting problems. Furthermore, the compact representations obtained from the fusion module can help the classification results narrow intra-distances and discriminate between distributions.

More concretely, we build our model with an instantiation of the proposed architecture on the top of a 50-layer pre-trained ResNeSt [49] (as shown in Table 1), which is a new ResNet [50] variant that integrates channel-wise attention with multi-path network representation. Compared to other state-of-the-art CNN models, the family of ResNeSt achieves higher accuracy and a better latency trade-off on the benchmark dataset. In this work, we remove the GAP and last FC layers and replace them with our modules. When the image inputs, the image size is 224×224; the backbone network constituted by original layers generates the output of feature maps with the size of 7×7×2048. Then the feature processing path branches off in the DSE block to produce two different feature map sizes (8×2048 and 49×2048). We stretch one of the feature maps with 8×2048 size to 49×2048 by exploiting FC for the following FBE layer. The dimension of outputs from the FBE layer falls to 1×2048, where the resulting vector is normalized with L2 normalization for the final classification. Furthermore, the new model can load the parameters of the ResNeSt-50 model that is pre-trained on the ImageNet dataset [51] to accelerate the training process, and all the parameters of the model can be trained end-to-end by backpropagation.

### 3.2. DSE Block

For brain tumor classification, since background and texture usually lack discriminative features in MRI images, the basic idea of our DSE block is to suppress the salient features’ impact based on background and texture details from global orderless information. Local spatial representation is a crucial element for classification, while the strategy of the DSE block is to randomly suppress the discriminative regions of the feature maps to guide network attention for a diverse set of relevant cues across the classes. Through superimposing these two suppression strategies, the network can capture more attentive features from input images.

#### 3.2.1. Global Orderless Feature Suppression

The encoding layer integrates the dictionary learning and residual encoding pipeline into a single CNN layer to provide global orderless information. Here we briefly present an overview of prior work for completeness. Let the input feature map size be c×h×w, which *c*, *h*, and *w* refer to the channel, height, and width. Then the visual descriptors are set to $X=\{x_{1},x_{2},\cdots ,x_{m}\}$, where m=h×w. The inherent codebook $D=\{d_{1},d_{2},\cdots ,d_{n}\}$ contains *n* learned codewords and the corresponding residual vector $r_{ij}$ can be written as $r_{ij}=x_{i}-d_{j}$, where i=1,⋯m and j=1,⋯n. Given an assigned weight $\omega_{ij}$ to each codeword for residual vector $r_{ij}$, the corresponding residual encoding model for every single codeword $d_{j}$ can be denoted by

(1) $$e_{j}=\sum_{i=1}^{m}e_{ij}=\sum_{i=1}^{m}\omega_{ij}r_{ij}$$

Considering the codeword ambiguity and the assigning weights for residual encoding model differentiation, the assigning weight $\omega_{ij}$ for the residual vector $r_{ij}$ can be represented as

(2) $$\omega_{ij}=\frac{\exp(-s_{j}{∥r_{ij}∥}^{2})}{\sum_{j=1}^{K}\exp(-s_{j}{∥r_{ij}∥}^{2})}$$

where $S=\{s_{1},s_{2},\cdots ,s_{m}\}$ denote learnable scaling factors for each codebook $d_{j}$.

**Figure 3:**
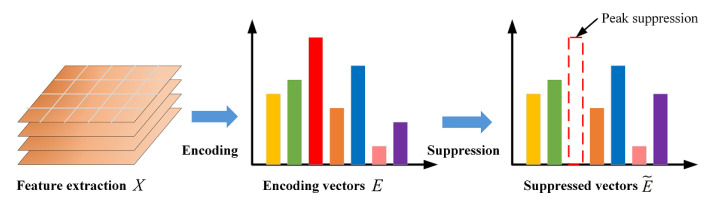
Overview of global orderless feature suppression strategy.

The encoding layer encodes a set of *K* residual encoding vectors $E=\{e_{1},e_{2}\cdots ,e_{n}\}$ of visual descriptors *X* to capture global orderless features. Here we extend the encoding layer with a suppression strategy, as shown in Figure 3. Let $P_{l}=\left\{p_{1},p_{2},\cdots p_{n}\right\}$ be the non-maximal map derived from *l*th residual encoding vector $E_{l}$, where $P\in R^{n}$ and l=1,⋯c. The form is generated as follow:

(3) $$P_{l}\left(j\right)=\left\{\begin{array}{c} \alpha ,\;\mathrm{if}E_{l}\left(j\right)=\max\left(E_{l}\right)\\ 1,\;\;\mathrm{otherwise} \end{array}\right.$$

where $\max(E_{l})$ denotes the maximum value of vector $E_{l}$ and α∈[0,1] is suppressing factor. We suppress the maximum value of each residual encoding vector such that:

(4) $${\tilde{E}}_{l}=P_{l}\circ E_{l}$$

where ∘ denotes the element-wise multiplication of two vectors. The intention behind the strategy of suppressing the salient feature is to weed out the effect of background and texture and consequently force the network to attend to more accurate discriminative features during propagation.

#### 3.2.2. Local Spatial Feature Suppression

**Figure 4:**
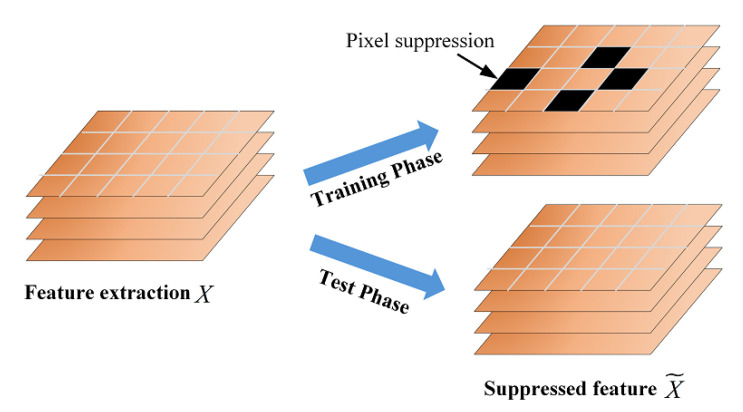
Overview of local spatial feature suppression strategy.

Consider that local spatial features bring focus to discriminative regions of a multi-class image. We expect the network to capture more subtle information from extracted feature maps by randomly suppressing active locations, as shown in Figure 4. As with prior visual descriptors *X*, here *X* also represents the feature map of a channel. $x_{i}$ is the *i*th pixel of *X*, where i=1,⋯m. We also let $P_{l}^{\prime }=\left\{p_{1}^{\prime },p_{2}^{\prime },\cdots p_{m}^{\prime }\right\}$ be the non-maximal map derived from the *l*th residual encoding vector $X_{l}$, where $P\in R^{m}$ and l=1,⋯c. The form is generated as follows:

(5) $$P_{l}^{\prime }\left(j\right)=\left\{\begin{array}{c} \beta ,\;\mathrm{if}X_{l}\left(j\right)=\max\left(X_{l}\right)\\ 1,\;\;\mathrm{otherwise} \end{array}\right.$$

where $\max(X_{l})$ denotes the maximum value of vector $X_{l}$ and β∈[0,1] is another suppressing factor. Then we randomly select feature map $X_{l}$ from all channels with a certain probability *r*, where *r* is a Bernoulli random variable. By suppressing the peak pixels randomly from each selected $X_{l}$, the network looks for the discriminative regions as an alternative to the suppressed representations in the image and outputs a modified vector ${\tilde{X}}_{l}$. The specific implementation of the suppression strategy is illustrated in Algorithm 1.
**Algorithm 1** The implementation of local spatial feature suppression.Here, the feature map is $X_{l}=\left\{x_{1},x_{2},\cdots ,x_{m}\right\}$, r∼Bernoulli(p) has *p* probability of being 1, *c* is the number of network channels, and ct is a random integer to control the number of loops.**for**l=1,⋯,c **do**    Initialize *r*;    **if** r==1 **then**        Initialize ct;        **for** i=1,⋯,ct **do**           ${\tilde{X}}_{l}=P^{\prime }\circ X_{l}$;    **else**        ${\tilde{X}}_{l}=X_{l}$;

Additionally, the local suppression strategy only works during the training phase. The whole feature map passes through the local suppression module without any suppression during the test phase so that all informative features can contribute to the final confidence score.

### 3.3. FBE Layer

**Figure 5:**
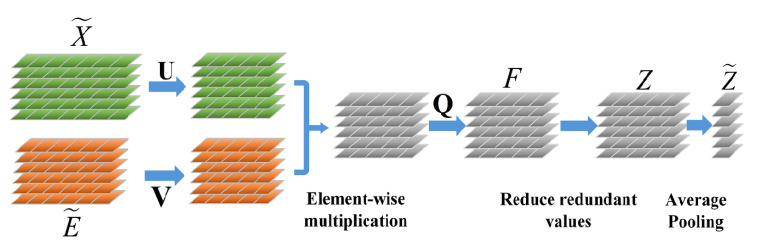
The architecture of the FBE module.

While classical fusion approaches such as concatenation based on first-order statistical information are effective at classification tasks, they have not matched the performance of bilinear pooling. The shortcoming of bilinear pooling is its redundancy, which may output high-dimensional features with many model parameters. The prior studies [44,52] inspired us to embed the FBE layer into our network to achieve a more effective fusion of the extracted features from different branches of the DSE Block. The architecture of the whole FBE Layer is illustrated in Figure 5.

As known from Section 3.2, let $\tilde{X}$ and $\tilde{E}$ represent the two extracted feature vectors with dimensionality *m* and *n*. $F=\{f_{1},f_{2},\cdots f_{o}\}$ is output vector and every pair of features is given by

(6) $$f_{i}={\tilde{X}}^{T}W_{i}\tilde{E}+b_{i}$$

where $W_{i}\in R^{m\times n}$ is a projection matrix for the output and $b_{i}$ is a bias. The number of parameters that are needed to learn is o×(m×n+1). Such a large number of parameters may cause high computational cost and overfitting risk. Li et al. [43] suggest a matrix factorization method to reduce the rank of matrix $W_{i}$. They rewrite the matrix as $W_{i}\;=\;U_{i}V_{i}^{T}$, where $U_{i}\in R^{m\times k}$, $V_{i}\in R^{n\times k}$, and $k\leq \min\left(m,n\right)$. Inspired by this idea, $f_{i}$ can be factorized as follows:

(7) $$f_{i}={\tilde{X}}^{T}U_{i}V_{i}^{T}\tilde{E}+b_{i}=1^{T}\left(U_{i}^{T}\tilde{X}\circ V_{i}^{T}\tilde{E}\right)+b_{i}$$

where $1^{T}\in R^{k}$ is an all-one column vector and denotes the element-wise multiplication of two vectors. Without losing generality, we reformulate 1 as $Q\in R^{k\times o}$ to decide the dimension of the output. Then we replace $b_{i}$ with $B\in R^{o}$, and redefine $U\in R^{m\times k}$ and $V\in R^{n\times k}$ to get the output feature *F* as follows:

(8) $$F=Q^{T}\left(U^{T}\tilde{X}\circ V^{T}\tilde{E}\right)+B$$

To further reduce the redundancy of the model and allow it to learn a compact representation in an end-to-end manner, we encode the *l*th vector $F_{l}$ into sparse code by exploiting the following solution of an optimization problem. Then we take the form as:

(9) $$Z_{l}=\underset{Z}{\arg\min}\left\{\frac{1}{2}{∥Z-F_{l}∥}_{2}^{2}+\lambda {∥Z∥}_{1}\right\}$$

Here, $Z_{l}\in R^{o}$ is the sparse code $F_{l}$ and l=1,⋯c. λ is a tuning parameter to adjust the model complexity. The L1-norm ${∥•∥}_{1}$ is used as a penalty term to impose the sparsity constraint on *Z*.

According to Iterative Shrinkage-Thresholding Algorithm (ISTA), we translate each iteration with a descent function to obtain the sparse code $Z_{l}$. Equation (9) is expanded as:

(10) $$Z_{l}=\underset{Z}{\arg\min}\left\{\sum_{i}\left[\frac{1}{2}{\left(Z\left(i\right)-F_{l}\left(i\right)\right)}^{2}+\lambda \left|Z(i)\right|\right]\right\}$$

where Z(i) and $F_{l}\left(i\right)$ are the *i*th components of *Z* and $F_{l}$ respectively. If Z(i)>0, Equation (10) follows the properties of quadratic and absolute value functions such that:

(11) $$Z_{l}\left(i\right)=\underset{Z}{\arg\min}\left\{\frac{1}{2}{\left(Z\left(i\right)-F_{l}\left(i\right)\right)}^{2}+\lambda Z\left(i\right)\right\}$$

where the derivative with respect to Z(i) is

(12) $$\frac{dZ_{l}\left(i\right)}{dZ(i)}=Z\left(i\right)-F_{l}\left(i\right)+\lambda$$

The minimum value of $Z_{l}\left(i\right)$ is given by

(13) $$Z_{l}\left(i\right)=\left\{\begin{array}{cc} F_{l}\left(i\right)-\lambda & ,F_{l}\left(i\right)\geq \lambda \\ F_{l}\left(i\right)+\lambda & ,F_{l}\left(i\right)\leq -\lambda \\ 0 & ,-\lambda <F_{l}\left(i\right)<\lambda \end{array}\right.$$

Based on the above discussion, we need to apply the single term solution method to each term of Equations (8) and (9), which yields:

(14) $$\left\{\begin{array}{c} Z_{l}=sign\left(F_{l}\right)\circ \max\left\{(\left|F_{l}\right|-\lambda ),0\right\}\\ F_{l}=Q^{T}\left(U^{T}{\tilde{X}}_{l}\circ V^{T}{\tilde{E}}_{l}\right)+B \end{array}\right.$$

At this point, we reduce the number of parameters and fuse the two input features simply by matrix factorization tricks. Finally, we compute our fusion representations $\tilde{Z}$ by using the average operation.

(15) $$\tilde{Z}=\mathrm{average}{\left\{Z_{l}\right\}}_{l=1}^{c}\;$$

## 4. Experimental Results and Analysis

In this section, we present experimental results and related analyses for the proposed brain tumor classification network. We first describe the datasets and implementation details for our network. Then we provide detailed qualitative and quantitative experiments on the two modules of our network to demonstrate the effectiveness of the proposed method mentioned in the paper. Finally, we report our network’s performance on public datasets and choose several state-of-the-art methods for comparison.

### 4.1. Experimental Setting

#### 4.1.1. Datasets

**Figure 6:**
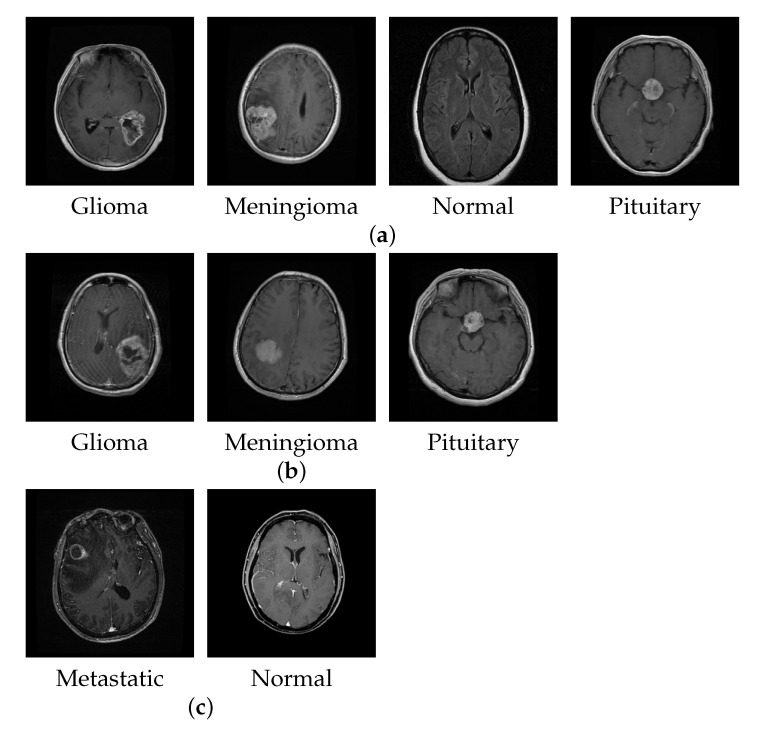
The examples of brain MRI slices in different datasets. (**a**) BT-4C, (**b**) BT-3C, (**c**) BT-2C.

For brain tumor classification, we collected available MRI datasets from three different repositories to perform a set of experiments, as shown in Table 2. The first dataset is from the Kaggle website [53] and consists of multiple publicly available dataset repositories. The dataset comprises 3264 brain MRI slices divided into four classes: meningioma, glioma, pituitary, and no tumor, with slice numbers 937, 926, 901, and 500, respectively. We named this dataset BT-4C for simplicity. The second dataset is Figshare [54], which contains 3064 T1-weighted contrast-enhanced images from 233 patients. This dataset comprises three brain tumor classes: meningioma, glioma, and pituitary tumor, with slice numbers 708, 1426, and 930, respectively. We named this dataset BT-3C. The third dataset was collected in a private repository from Changzhou No.2 People’s Hospital in China in 2021. All image slices consisted of plain and dynamic enhanced MRI for metastatic tumor detection. We cleaned the data by approaching a doctor who examined all the MRIs manually. This dataset comprises 1109 slices, out of which 495 slices are metastatic tumor and the remaining 614 slices are normal without tumor. We named this dataset BT-2C due to it containing two classes. Figure 6 shows the examples of MRI brain images in BT-4C, BT-3C, and BT-2C datasets. In Section 4.2, the dataset is used for qualitative and quantitative analysis with the subdivisions of a training set, a validation set, and a test set. Of the dataset, 70% was for training, 15% was for validation, and the rest 15% was for testing. In Section 4.3, we conducted experiments with different partition ratios and cross-validation schemes to further test the performance of our proposed method. Additionally, for fair comparisons with the state-of-the-art works, we followed the same settings in the literature.

#### 4.1.2. Implementation Details

To fairly compare the different methods with and without novelties mentioned above, we used an input image resolution of 224×224 in all experiments. At the default setting of the hyperparameters, the optimal parameters were learned with an Adam optimizer. The initial learning rate of the experiments was 1e-4, which halve decays when the loss stops improving for two consecutive epochs during training. The learning rate of added and modified layers was ten times higher than that of pre-trained layers. We applied the Pytorch environment to perform all the experiments with NVIDIA Quadro RTX4000 GPU.

#### 4.1.3. Evaluation Metrics

Furthermore, we adopt the performance evaluation metrics to evaluate the generalization ability of the proposed network, i.e., precision, recall, specificity, F1 score, accuracy, and area under the curve (AUC). The mathematical expressions are defined as follows.

(16) $$precision=\frac{TP}{TP+FP}$$

(17) $$recall=\frac{TP}{TP+FN}$$

(18) $$specificity=\frac{TN}{TN+FP}$$

(19) $$F1score=2\times \frac{recall\times precision}{recall+precision}$$

(20) $$accuracy=\frac{TP+TN}{TP+TN+FP+FN}$$

TP (true positive), means the correct prediction of the positive class. FP (false positive), means the incorrect prediction of the positive class. Similarly, TN (true negative) is an outcome that correctly predicts the negative class, while FN (false negative) is an outcome that incorrectly predicts the negative class. Hence, precision is the proportion of true positives in the total of positive predictions, recall is the proportion of positive cases correctly predicted, specificity is the proportion of negative cases correctly predicted, F1 score is the weighted average of precision and recall, and accuracy is the proportion of correctly classified observations.

### 4.2. Qualitative and Quantitative Analysis

In this section, we design a series of experiments to test the effectiveness of the proposed components in the network for detailed analysis. Each one was compared with a similar network as a baseline to highlight the importance of the DSE block and the FBL layer.

#### 4.2.1. Ablation Experiments

We conducted all ablation experiments on dataset BT-4C by using ResNeSt-50 as a backbone to highlight the importance of each component in our network. Here, we constructed a similar network as a baseline for ablation investigation. First, we retained the dual path from the proposed network to extract global and local features without any suppression strategy, naming this “Dual-path” as a benchmark of DSE. Then we adopted a concatenation layer for fusion as a substitute to FBE.

Table 3 provides the accuracy rates for various combinations of proposed methods. Applying the DSE block in the ResNet-50 backbone improves the accuracy from 95.51% to 96.94%. Using the FBE layer results in a performance improvement of 0.61% (from 95.51% to 96.12%). We note that the baseline network modified without suppression strategy leads to few accuracy improvements, as the feature extractions presumably contain influence factors. Thus, the DSE block cooperating with the FBE layer caused an immediate effect on performance improvement.

#### 4.2.2. Effect of DSE

**Figure 7:**
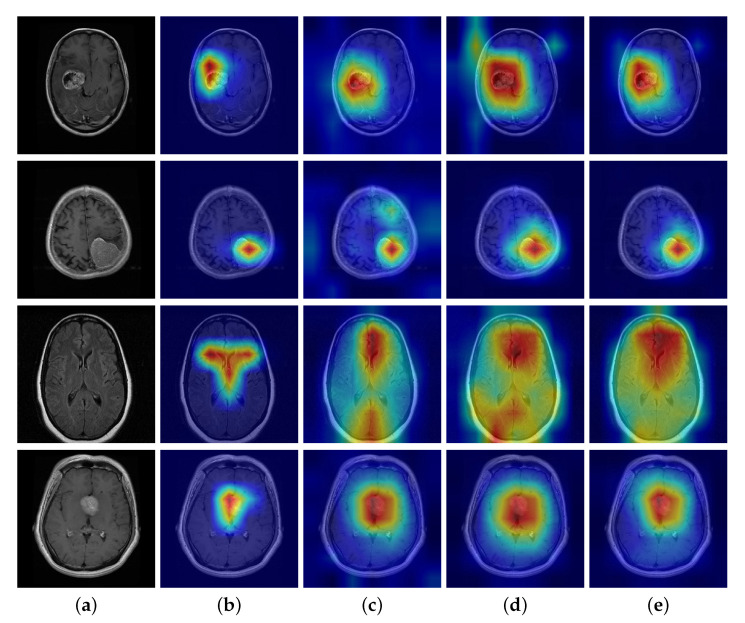
Class activation map (CAM) comparison with and without our modules in dataset BT-4C. *Top* to *bottom*: glioma, meningioma, normal, pituitary. (**a**) Original image, (**b**) CAM with GS module, (**c**) CAM without GS module, (**d**) CAM with LS module, (**e**) CAM without LS module.

The DSE block uses an adaptive strategy harmony with the characteristics of brain tumor MRIs to guide the network in suppressing the interfering factors and extracting more informative features. The effect of the global orderless feature suppression (GS) strategy is quite different from that of the local spatial feature suppression (LS) strategy. In an attempt to clarify the working mechanism of each module, here we present a comparison between our method and the baseline by class activation map (CAM) observation, as shown in Figure 7.

Firstly, the GS module was introduced in CNN separately for discriminative localization using the GAP. The global feature is a set of abstract orderless encodes, in which some clues can unfold from CAM. Figure 7a illustrates that the network with GS prunes the CAM region compared to Figure 7b without GS. Specifically, when applying the BS strategy, the salient area of the CAM is clear even if there is no tumor in the MRI. In contrast, without BS, there is bit diffusion or worse for no-tumor MRIs. The explanation of the difference is that the network works the way we intended it to, being more concerned with distinct discriminative features from global encodes than background or texture ones.

Secondly, we processed the LS module in the same way to generate the CAM. As the local feature is an aggregation of spatial order information, it seemed more suitable for CAM to interpret what parts of the image are of interest to the network. Figure 7c,d shows that the LS module achieves an effect opposite to that of the GS. When applying the LS strategy, the CAM displays an extended salient area where the network pays more attention to the tumor and its associated parts. In contrast, without LS, there is a limited set of the salient region of spatial locations. This implies that the LS module helps the network excavate a more informative area, which is expected to improve classification performance.

The definition of all brain tumor types depends on the tumor size and the related parts involved in the brain [55]. That explains why the DSE block triggers two opposite effects, as the global orderless feature focuses on the tumor itself and the local spatial one concentrates on the associated part of the brain.

Here, we conduct experiments with different values of suppressing factor α and β in Equation (3)–(5) to demonstrate our inference. Table 4 shows the results of parameter sensitivity on dataset BT-4C, that is, how these two parameters influence the model classification performance. One can see that the network achieves better performance when α and β are kept as small values than it does without using the DSE block (α=β=1), which indicates that the DSE block contributes to brain tumor classification. The values α=0 and β=0.1 especially lead to the highest accuracy.

#### 4.2.3. Effect of FBE

**Figure 8:**
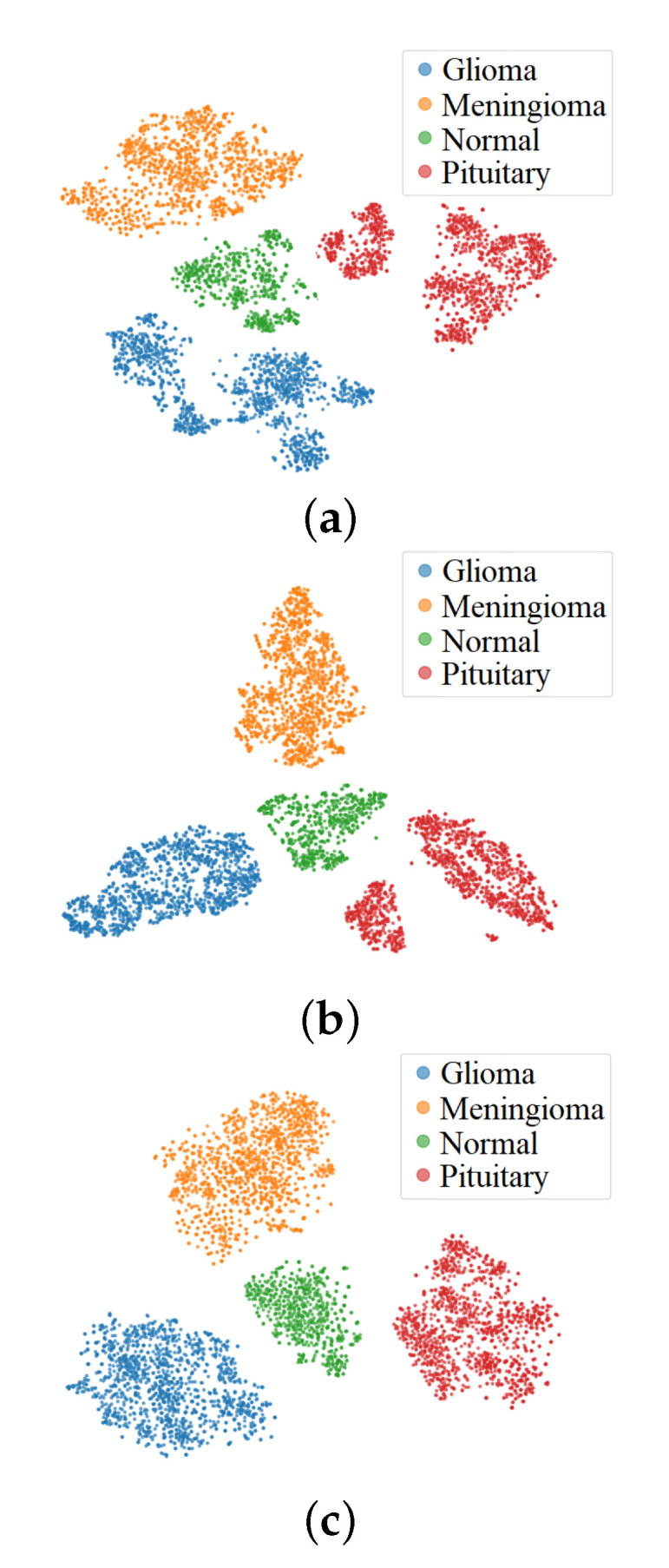
Visualization of feature distribution of three classification models on dataset BT-4C. (**a**) ResneSt, (**b**) ResneSt + bilinear pooling, (**c**) ResneSt + FBE.

The FBE layer uses a fusion technique based on bilinear pooling from a factorized sparse coding perspective. Since feature distributions that output from two branches of the DSE block may vary dramatically, the effect of FBE is to fully capture complex associations between these two types of features and balance their contributions for sufficient expressiveness of the network. To analyze the effect of the FBE, we randomly choose 80% of images from dataset BT-4C as a training set and collected feature representations before classification layers. Then we plotted the feature distributions using the t-SNE [56] to observe class variance, as shown in Figure 8. The plots suggest that the FBE obtains a better visualization effect in separating the inter-class and clustering the intra-class.

Since brain tumor type recognition from different MRIs requires experts to distinguish and annotate them according to professional knowledge, brain tumor classification is a fine-grained recognition task. That explains why the FBE layer is more effective, since it is a fusion technique based on fine-grained features. We designed experiments to compare FBE with other methods based on bilinear pooling and observed the results on a brain tumor classification task according to different options. Here, we used the ResNeSt-50 network as the backbone and removed the layers after “layer4”. The two groups of features for fusion are the output of “layer4” and its copies. We then applied each method in turns to obtain representations, followed by an FC layer. Table 5 shows the accuracy results of the listed methods, where the option *o* is output dimension, and *k* is the rank of factorized matrixes (such as U and V in Equation (14)).

We can see that our FBE method further improves the accuracy compared with other methods. When we set the parameter of FBE to k=2 and o=2048, its performance achieves 96.94% on the BT-4C dataset. It was also found that compact bilinear pooling [32] and low-rank bilinear pooling [52] can immensely reduce the number of parameters from the output vector to produce compact representations. On this basis, FBE aims to further improve discrimination by using comparable parameters for higher accuracies.

### 4.3. Performance Comparison

In this section, we choose several representative networks and state-of-the-art methods to compare the performance of their brain tumor classification with our method. The test results of these methods were obtained from presented literature or the model was retrained using code resources provided by public repositories. We follow the same conditions to train our model.

#### 4.3.1. Performance on Dataset BT-4C

**Figure 9:**
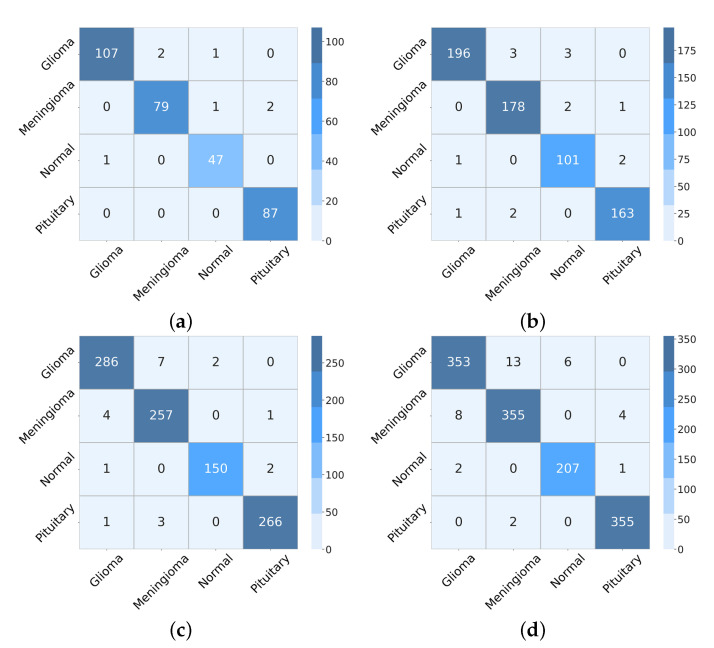
The confusion matrixes derived from the proposed model on BT-4C with different partition ratios. (**a**) 90:10, (**b**) 80:20, (**c**) 70:30, (**d**) 60:40.

**Figure 10:**
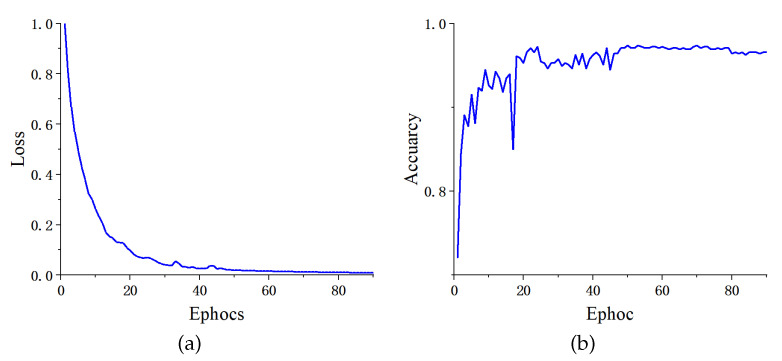
The curves of the training process on dataset BT-4C. (**a**) Training loss, (**b**) validation accuracy.

We analyzed the proposed network on dataset BT-4C in the previous section. Here, we use more transverse tests to analyze the generalization performance of the proposed method. Table 6 details the evaluation metrics of classification performance with different partition ratios between training and testing sets from 90:10 to 60:40. With the variation of the partition ratio of training and testing set, the evaluation metrics for each category obtained by the proposed network are barely degraded. Intuitive suggests that our method still learns the key discriminative features even under the limitation of training data, thus maintaining classification performance at a high level. The confusion matrices illustrated in Figure 9 further prove the positive roles of the proposed method in this classification task from the actual and predicted labels. The accuracy reaches 0.9786, 0.9770, 0.9786, and 0.9732 with partition ratios of 90:10, 80:20, 70:30, and 60:40 between training and testing sets, respectively. Figure 10 takes the ratio of 80:20 as an example to illustrate the curves of the training process.

**Figure 11:**
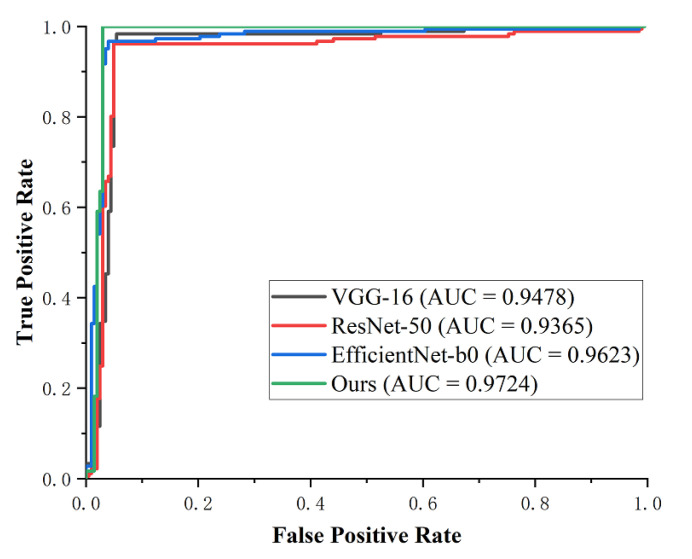
Comparison of the ROC curves of different methods.

We then compared the classification performance of the proposed network with that of three pre-trained CNN models, i.e., VGG-16 [57], ResNet-50 [50], and EfficientNet-B0 [58]. They all performed the same experiments with a partition ratio of 80:20 and applied the same transfer learning and fine-tuning strategy. Table 7 gives the classification results of macro-average evaluation metrics. Our method shows the best performance, exceeding the other three models on each metric, where our method obtains 2.14%, 1.99%, and 1.53% higher accuracy than VGG-16, ResNet-50, and EfficientNet-B0, respectively. To analyze the value of each model more intuitively, we plotted the ROC curves for each experiment with the same coordinates, as shown in Figure 11. The accuracy of VGG-16 is lower than that of Resnet-50, while the AUC is higher, implying that VGG-16 had a better diagnostic value than Resnet-50 in this experiment. EfficientNet-B0 achieves an AUC of 0.9623 and performs better than VGG-16 and ResNet-50. Our method shows the best AUC of 0.9724 among the models, proving its superior performance for the classification task.

#### 4.3.2. Performance on Dataset BT-3C

**Figure 12:**
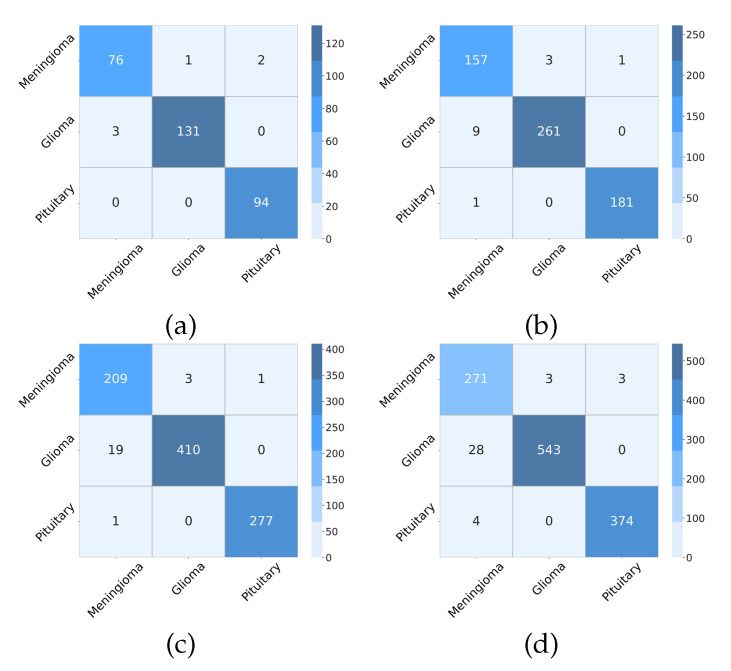
The confusion matrixes derived from the proposed model on BT-3C with different partition ratios. (**a**) 90:10, (**b**) 80:20, (**c**) 70:30, (**d**) 60:40.

**Figure 13:**
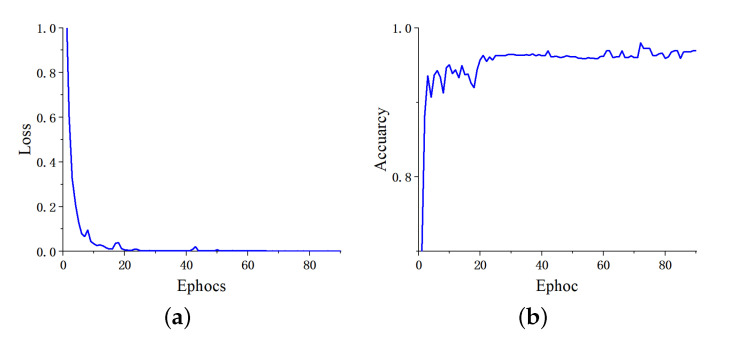
The curves of the training process on dataset BT-3C. (**a**) Training loss, (**b**) validation accuracy.

To revisit the generalization of the proposed method, we applied our network to another BT-3C dataset with the same experimental setting as Section 4.3.1. Table 8 shows the evaluation metrics of classification performance for each of the three categories. As the proportion of training data in the total dataset falls while the testing data grows accordingly, the performance of the proposed network showed an insignificant decline. The reason behind the decline lies in distribution inconsistency between training and testing datasets. However, each evaluation metric still holds steady at a relatively high percent due to the proposed network with powerful learning and generalizing ability. As illustrated in Figure 12, we can obtain more direct results from the confusion matrices. When the partition ratio is 90:10, the proposed network attains a classification accuracy of 0.9805 with an ROC of 0.9687. When the partition ratio is 80:20, the accuracy is 0.9772, and the ROC is 0.9634. Figure 13 also shows the curves of the training process. At a ratio of 70:30, we get an accuracy of 0.9739 with an ROC of 0.9565. Even if the partition ratio reaches 60:40, the accuracy remains at 0.9690 while the ROC is 0.9593.

As described in Table 9, we can make an extended comparison to some extent of the results produced by our proposed method with the state-of-art ones in the literature using the same dataset. Studies [2,59] adopted traditional methods to extract global statistical features, then classified them by classifiers to achieve the final decision. Studies [60,61] deployed their model using CapsNet [62] to vary the feature maps in the convolutional layer. Studies [26,63,64] combined deep CNN and machine learning classifiers for brain tumor classification with a series of fine-tuning operations. Studies [16,22,28] used multiple models to grab more information for high accuracy in the classification task. The literature shows improvements in network architecture and training tricks, but does not elaborate on the rationale for extracting features. Our work is the pioneer study in exploring the essence of brain tumor features with the proposed method. As a result, the accuracy we obtained is superior to that of other existing models.

#### 4.3.3. Performance on Dataset BT-2C

**Figure 14:**
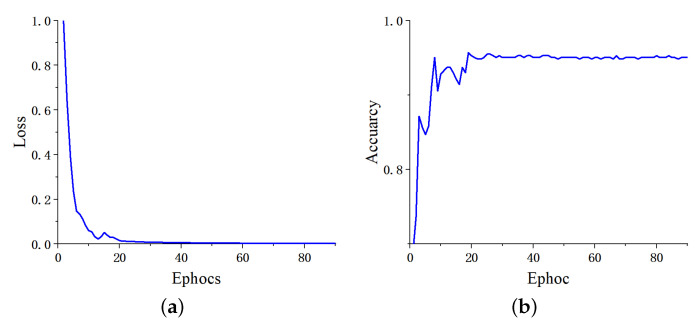
The curves of the training process on dataset BT-2C. (**a**) Training loss, (**b**) validation accuracy.

The dataset BT-2C was acquired to validate the generalization of the proposed network in metastatic tumor detection. Unlike other tumor classification tasks, metastatic tumors are sometimes too small to be detected. Even a specialist needs to comprehensively study different modalities of MRI scans (such as plain and dynamic enhanced MRI) for make a final decision. Given the limited samples in the dataset, we introduced cross-validation to evaluate the performance of our method. The experiment conducted a standard cross-validation procedure of three-fold, five-fold, and ten-fold. In the comparison, we used the same pre-trained CNN models such as VGG-16 [57], ResNet-50 [50], and EfficientNet-B0 [58]. Table 10 describes the average results of each evaluation metric for the two classes: normal and abnormal (metastatic). Figure 14 presents an example to depict the curves of the training process in five-fold cross-validation. After integrated analysis of each test result, our proposed method shows clear superiority to other pre-trained models.

**Figure 15:**
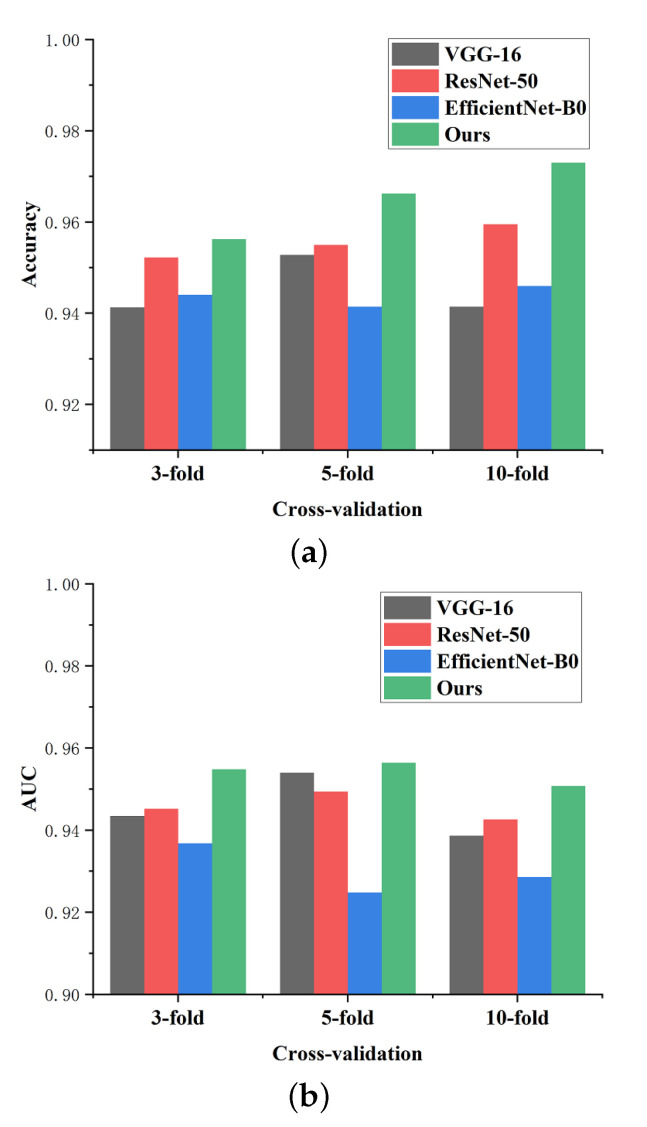
Comparison of cross-validation performance for different methods on dataset BT-2C. (**a**) Accuracy comparison, (**b**) AUC comparison.

Figure 15 depicts the average results of accuracy and AUC for each method under the different cross-validation mechanisms. The results reflect that our model can achieve persistent optimal classification performance under any cross-validation mechanism. Specifically, when adopting three-fold cross validation, the accuracy of the proposed network is 0.9508 and the AUC is 0.9490. We achieve a classification accuracy of 0.9685 with a ROC of 0.9638 under five-fold cross-validation. When using ten-fold cross validation to evaluate the performance, the accuracy is 0.9820 and the ROC is 0.9530.

## 5. Conclusions

This paper has proposed a novel method based on a pre-trained model for brain tumor classification that synergizes a factorized bilinear network with a dual suppression strategy. The proposed DSE block uses two methods to find the exact features that represent the differences between similar classes. One method is to refine global orderless features for more valuable clues by reducing the impact of negative ones. The other is to shift attention to salient regions from local spatial features. An appropriate fusion layer named FBE is then used to generate compact and discriminative representations. Finally, we port the two components into a pipeline where end-to-end learning is achieved. As a result, our proposed method demonstrates excellent performance in brain tumor classification on three datasets, outperforming other state-of-the-art methods without any data augmentation. Moreover, we conducted experiments to explore the connection between the character of brain tumor MRIs and the convolutional feature map. In the future, we will further analyze information on MRIs from different modalities and relationships between them by exploiting deep CNNs, in order to help specialists complete diagnoses quickly.

## Figures and Tables

**Table 1:** The architectures for adopting 50-layer pre-trained ResNeSt. The input image size is 224×224.

Layer Name	Filter Type		Number	Output Size
layer0	conv1	$\left[\begin{array}{c} 3\times 3,32,\mathrm{stride}\;2\\ 3\times 3,32\\ 3\times 3,64 \end{array}\right]$	×1	112×112×64
	downsample	3×3maxpool,stride2		
layer1	conv1	1×1×64	×3	56×56×128
	conv2	$\left[\begin{array}{c} 3\times 3,128\\ 1\times 1,32\\ 1\times 1,128 \end{array}\right]$		
	conv3	1×1,256		
	downsampl	1×1averagepool,stride2	×1	
		1×1,256		
layer2	conv1	1×1×128	×4	28×28×512
	conv2	$\left[\begin{array}{c} 3\times 3,256\\ 1\times 1,64\\ 1\times 1,256 \end{array}\right]$		
	conv3	1×1,512		
	downsampl	2×2averagepool,stride2	×1	
		1×1,512		
layer3	conv1	1×1×256	×6	14×14×1024
	conv2	$\left[\begin{array}{c} 3\times 3,512\\ 1\times 1,128\\ 1\times 1,512 \end{array}\right]$		
	conv3	1×1,1024		
	downsampl	2×2averagepool,stride2	×1	
		1×1,1024		
layer4	conv1	1×1×512	×3	7×7×2048
	conv2	$\left[\begin{array}{c} 3\times 3,1024\\ 1\times 1,256\\ 1\times 1,1024 \end{array}\right]$		
	conv3	1×1,2048		
	downsampl	2×2averagepool,stride2	×1	
		1×1,2048		
DSE Block	Section 3.2		×1	49×2048
FBE Layer	Section 3.3			1×2048
Classification	1×1,FC			n classes

**Table 2:** The main characteristics of the three datasets.

Dataset	Class	Number	Resolution	Format
BT-4C	Glioma	937	512×512,	.jpg
	Meningioma	926	350×350,	
	Normal	500	630×630,	
	Pituitary	901	etc.	
BT-3C	Glioma	708	512×512	.mat
	Meningioma	1426		
	Pituitary	930		
BT-2C	Metastatic	495	512×512	.jpg
	Normal	614		

**Table 3:** Ablation analysis on the dataset BT-4C. Our DSE block and FBE layer provide progressive improvements over the baseline.

Methods	Accuracy (%)
ResNeSt-50	95.91
ResNeSt-50 + Dual-path + Concat	95.91
ResNeSt-50 + DSE + Concat	97.14
ResNeSt-50 + Dual-path + FBE	96.94
Ours	**97.96**

**Table 4:** Accuracy with different combinations of suppressing parameters α and β on dataset BT-4C.

	β	0	0.1	0.2	1
α					
0		97.55	97.96	97.35	96.73
0.1		97.35	97.35	97.14	96.73
0.2		96.94	96.94	96.73	96.53
1		96.72	96.53	96.53	96.33

**Table 5:** Overall accuracies of different fusion methods on dataset BT-4C.

Method	Options	Accuracy (%)
Bilinear pooling [32]	-	96.86
Compact bilinear pooling [42]	o=8000	95.56
	*o* = 16,000	95.71
Low-rank bilinear pooling [52]	k=1,o=2048	96.32
	k=2,o=1024	96.17
	k=4,o=512	96.17
FBE	k=1,o=2048,λ=0.01	96.63
	k=2,o=1024,λ=0.01	**96.94**
	k=4,o=512,λ=0.01	96.48

**Table 6:** Classification performance of the proposed method on dataset BT-4C.

Partition Ratio	Class	Precision	Recall	Specificity	F1 Score
90:10	Glioma	0.9907	0.9727	0.9954	0.9817
	Meningioma	0.9753	0.9634	0.9918	0.9693
	Normal	0.9592	0.9792	0.9928	0.9691
	Pituitary	0.9775	1.0000	0.9917	0.9886
80:20	Glioma	0.9899	0.9703	0.9956	0.9800
	Meningioma	0.9727	0.9834	0.9894	0.9780
	Normal	0.9528	0.9712	0.9909	0.9619
	Pituitary	0.9819	0.9819	0.9938	0.9819
70:30	Glioma	0.9795	0.9695	0.9912	0.9744
	Meningioma	0.9625	0.9809	0.9861	0.9716
	Normal	0.9868	0.9804	0.9976	0.9836
	Pituitary	0.9888	0.9852	0.9958	0.9870
60:40	Glioma	0.9725	0.9489	0.9893	0.9605
	Meningioma	0.9595	0.9673	0.9851	0.9634
	Normal	0.9718	0.9857	0.9945	0.9787
	Pituitary	0.9861	0.9944	0.9947	0.9902

**Table 7:** Classification performance of the proposed method on dataset BT-4C.

Method	Precision	Recall	Specificity	F1 Score	Accuracy
VGG-16	0.9522	0.9533	0.9851	0.9526	0.9556
ResNet-50	0.9544	0.9556	0.9856	0.9549	0.9571
EfficientNet-B0	0.9608	0.9623	0.9870	0.9615	0.9617
Ours	**0.9743**	**0.9767**	**0.9924**	**0.9755**	**0.9770**

**Table 8:** Classification performance of the proposed method on dataset BT-3C.

Partition Ratio	Class	Precision	Recall	Specificity	F1 Score
90:10	Meningioma	0.9620	0.9620	0.9868	0.9620
	Glioma	0.9924	0.9776	0.9942	0.9850
	Pituitary	0.9792	1.0000	0.9906	0.9895
80:20	Meningioma	0.9401	0.9752	0.9779	0.9573
	Glioma	0.9886	0.9667	0.9912	0.9775
	Pituitary	0.9945	0.9945	0.9977	0.9945
70:30	Meningioma	0.9127	0.9812	0.9717	0.9457
	Glioma	0.9927	0.9557	0.9939	0.9739
	Pituitary	0.9964	0.9964	0.9984	0.9964
60:40	Meningioma	0.8944	0.9783	0.96628	0.9345
	Glioma	0.9945	0.9510	0.9954	0.9722
	Pituitary	0.9920	0.9894	0.9965	0.9907

**Table 9:** Comparison between the proposed and existing state-of-the-art methods.

Reference	Method	Accuracy (%)
Cheng et al. [59]	BoW + SVM	91.28
Ismael et al. [2]	DWT + Gabor	91.90
Afshar et al. [60]	CapsNet	90.89
Phaye et al. [61]	Diverse CapsNet	95.03
Anaraki et al. [26]	CNN + Genetic Algorithm	94.20
Kaplan et al. [63]	nLBP + KNN	95.56
Deepak and Ameer [64]	Freeze GoogLeNet-inception-4e + SVM	95.44
Noreen et al. [28]	Inception-v3 + Ensemble Learning	94.34
Jyostna et al. [22]	Two-channel DNN	95.23
Badža et al. [16]	Four-layer CNN	97.39
Proposed	ResNeSt-50 + DSE + FBE	**98.02**

**Table 10:** Classification performance of the proposed method on dataset BT-2C.

Cross-Validation	Method	Class	Precision	Recall	Specificity	F1 Score
3-fold	VGG-16	Metastatic	0.9371	0.9577	0.9212	0.9472
		Normal	0.9471	0.9213	0.9577	0.9338
	ResNet-50	Metastatic	0.9401	0.9751	0.9242	0.9573
		Normal	0.9683	0.9243	0.9751	0.9458
	EfficientNet-B0	Metastatic	0.9228	0.9801	0.9000	0.9506
		Normal	0.9738	0.9000	0.9801	0.9354
	Ours	Metastatic	0.9672	0.9528	0.9606	0.9599
		Normal	0.9437	0.9606	0.9528	0.9520
5-fold	VGG-16	Metastatic	0.9433	0.9709	0.9314	0.9569
		Normal	0.9645	0.9314	0.9709	0.9477
	ResNet-50	Metastatic	0.9472	0.9709	0.9363	0.9589
		Normal	0.9647	0.9363	0.9709	0.9503
	EfficientNet-B0	Metastatic	0.9084	0.9917	0.8824	0.9482
		Normal	0.9890	0.8824	0.9917	0.9326
	Ours	Metastatic	0.9710	0.9667	0.9657	0.9687
		Normal	0.9612	0.9657	0.9667	0.9633
10-fold	VGG-16	Metastatic	0.9750	0.9207	0.9688	0.9469
		Normal	0.9034	0.9688	0.9207	0.9348
	ResNet-50	Metastatic	0.9466	0.9841	0.9271	0.9650
		Normal	0.9781	0.9271	0.9841	0.9518
	EfficientNet-B0	Metastatic	0.9197	0.9921	0.8855	0.9543
		Normal	0.9889	0.8855	0.9921	0.9338
	Ours	Metastatic	0.9690	0.9841	0.9584	0.9765
		Normal	0.9788	0.9584	0.9841	0.9683

## Data Availability

The data sets generated during and/or analyzed during the current study are available from the corresponding author on reasonable request.
